# Variations over time in proximal femoral strength in young adult men are not explained by areal bone mineral density alone

**DOI:** 10.1093/jbmrpl/ziaf136

**Published:** 2025-08-16

**Authors:** Shoaib Attar, Hanna Isaksson, Lars Jehpsson, Bjorn E Rosengren, Magnus K Karlsson, Lorenzo Grassi

**Affiliations:** Department of Biomedical Engineering, Lund University, Box 118, 221 00 Lund, Sweden; Department of Biomedical Engineering, Lund University, Box 118, 221 00 Lund, Sweden; Clinical and Molecular Osteoporosis Research Unit, Department of Orthopedics and Clinical Sciences, Lund University, Box 118, 221 00 Lund, Sweden; Department of Orthopedics, Skane University Hospital (SUS), 205 02, Malmö, Sweden; Clinical and Molecular Osteoporosis Research Unit, Department of Orthopedics and Clinical Sciences, Lund University, Box 118, 221 00 Lund, Sweden; Department of Orthopedics, Skane University Hospital (SUS), 205 02, Malmö, Sweden; Clinical and Molecular Osteoporosis Research Unit, Department of Orthopedics and Clinical Sciences, Lund University, Box 118, 221 00 Lund, Sweden; Department of Orthopedics, Skane University Hospital (SUS), 205 02, Malmö, Sweden; Department of Biomedical Engineering, Lund University, Box 118, 221 00 Lund, Sweden

**Keywords:** osteoporosis, hip biomechanics, DXA, 2D-to-3D reconstruction, proximal femoral strength

## Abstract

The decline in areal BMD (aBMD) in men may start as early as age 19, but it is debated whether proximal femoral strength follows the same pattern. We measured FN aBMD in a cross-sectional cohort of 1101 men aged 18-28 yr with two-dimensional DXA. Body height, weight, jump height, grip strength, and maximum knee extension torque muscle strength were measured with standard tests, after which BMI was calculated. We adopted a validated method to automatically obtain three-dimensional finite element (FE) models from 2D DXA images and predict proximal femoral strength. We compared FN aBMD and FE-predicted proximal femoral strength as a function of age. Linear mixed-effect models (LMEs) were used to evaluate the association between age (whole years), FN aBMD, FN area, jump height, grip strength, maximum knee extension torque, and FE-predicted proximal femoral strength. A negative correlation (*r* = −0.10, *p* = .001) was observed between FN aBMD and age between ages 18 and 28; no statistically significant correlation (*r* = −0.02, *p* = .55) between age and FE-predicted proximal femoral strength was found. There was a positive correlation between FE-predicted proximal femoral strength and the subjects’ height (*r* = 0.22, *p* < .001) and weight (*r* = 0.35, *p* < .001). Higher jump height (*r* = 0.13, *p* < .001), grip strength (*r* = 0.23, *p* < .001), and maximum knee extension torque (*r* = 0.31, *p* < .001) were associated with higher FE-predicted proximal femoral strength. Femoral neck aBMD, FN area, and body height explained ~78% of the variations in proximal femoral strength in the LMEs. The inter-subject variations in proximal femoral strength were associated with FN aBMD, FN area, body height, and higher outcomes in jump height, grip strength, and maximum knee extension torque.

## Introduction

Osteoporosis is associated with low bone strength and a high fracture risk.^1^ In 2010, ~3.5 million patients in Europe sustained an incident fragility fracture. The cost of treating fragility fractures in Europe was estimated to be around €37 billion, and 22 million women and 5.5 million men were estimated to have osteoporosis.^2^^,^^3^ With an aging population, the number of fragility fractures is projected to increase further.^2^^,^^3^ Hence, it is essential to reduce the prevalence of osteoporosis. Peak bone mass (PBM) is defined as the highest amount of bone mass during a person’s lifetime^4^ and is typically reached at the end of skeletal maturity during the second decade of life. It is usually estimated based on areal BMD (aBMD) measurements from DXA scans.^5^ Peak bone mass is the most important modifiable determinant of lifelong skeletal health; a 10% increase in PBM can delay the onset of osteoporosis by ~13 yr,^6^ and exercise and lifestyle interventions can provide significant improvements to PBM.^7^

Peak hip aBMD occurs in healthy men between the ages of 16 and 22 yr.^5^^,^^8^^-^^11^ After the peak, there may be as much as ~5% decrease in aBMD by age 28.^5^^,^^10^^,^^11^ Age when PBM is reached, as well as the magnitude of the subsequent loss in aBMD, can be influenced by genetics and lifestyle factors such as nutrition and exercise.^4^ Common tests, such as grip strength, jump height, and isokinetic torque measurements of the lower leg, which are influenced by both genetics and physical activity, have also been shown to correlate with aBMD,^12^^-^^14^ as has the amount of exercise.^8^^,^^15^^-^^17^ Subjects with high physical activity levels have up to 13% higher FN aBMD compared to sedentary subjects.^8^ However, these large differences in aBMD have predominantly been found when comparing professional athletes to the community-dwelling population.^18^^-^^20^ Thus, it is still debated to what extent variations in physical activity contribute to the variation in aBMD in community-dwelling healthy adults.

Besides, the key parameter to assess skeletal health and the bone’s resistance to trauma is bone strength.^21^ Areal BMD is only one of the many determinants of bone strength, and it proved to be alone only a moderate surrogate of bone strength.^22^^,^^23^ Other factors of importance for bone strength include the amount of minerals in the skeleton, bone size, bone shape, and skeletal microstructure.^21^^,^^24^^,^^25^ It is therefore important to clarify whether aBMD and bone strength their reach peak value at the same age and if they follow the same pattern with increasing age. The advantage of using aBMD is that it is readily available and allows measurements in large populations using a reliable and standardized method with a reasonable fracture-predictive ability.^26^ However, a reduction in DXA-estimated aBMD may be the result of a decline in BMC in a non-growing bone, an increase in bone size in a bone with no changes in BMC, or a combination of changes in bone size and BMC.^5^ Hence, using aBMD to estimate bone strength can lead to inaccurate conclusions. This has been abundantly shown in elderly subjects,^22^^,^^23^^,^^27^^-^^29^ and may be even more relevant for subjects who have not reached full skeletal maturity. In the latter subjects, a decline in aBMD because of a decrease in BMC would lead to a weaker skeleton, while a decrease in aBMD because of an increase in bone size during growth would increase the bending strength of the bone. This has been shown for the proximal radius in elderly subjects,^7^^,^^30^ but similar mechanistic effects are expected at the hip in young growing individuals as well. Thus, a reduction in the amount of minerals in a bone may not necessarily result in reduced bone strength. Studies have shown that bone size in young adult men increases from age 19 to 28.^5^^,^^8^^,^^9^ This leads to uncertainty when estimating bone strength using aBMD at these ages and challenges the notion that femoral strength starts decreasing with aBMD after reaching PBM.

Three-dimensional (3D) subject-specific finite element (FE) models are more accurate in predicting bone strength than aBMD.^31^^-^^34^ Previous studies have looked at variations of FE-predicted bone strength with age but only focused on the fourth and fifth decade of life.^35^ To the best of our knowledge, no study has specifically evaluated 3D FE-predicted bone strength in the period around and immediately following the occurrence of PBM and the onset of decline in aBMD.^5^^,^^17^ One reason could be that 3D subject-specific FE models are typically built from CT scans, which have a high dose of radiation and high operational costs.^36^ Our research group has previously developed and validated a method to reconstruct 3D anatomies and generate 3D subject-specific FE models of the proximal femur from two-dimensional (2D) DXA images,^37^ with an accuracy comparable to conventional CT-based FE models,^34^^,^^38^ and superior ability to predict incident hip fractures compared to aBMD in prospective clinical cohorts.^22^^,^^29^

The aim of this study was to evaluate FN aBMD and FE-predicted proximal femoral strength in a cross-sectional cohort of community-dwelling men aged 18-28 yr, in relation to age and factors of importance for the variance in proximal femoral strength. We hypothesized that the morphological changes occurring in growing skeletons positively affect proximal femoral strength and can counteract the decline in aBMD that starts immediately after reaching PBM. We also hypothesized that different levels of physical performance can explain variations in proximal femoral strength both across individuals and within different age groups. We tested our hypotheses on a population-based cohort of young adults that was specifically designed to investigate PBM.^5^

## Materials and methods

The study was conducted on the MrPeak cohort.^5^ MrPeak is a cross-sectional population-based cohort that includes 1101 community-living men aged 18-28 yr residing in Malmö region, Sweden. The men were evaluated between 2006 and 2012. The cohort has been previously described in detail^5^ and is only briefly summarized here. The body height of these men was measured with a Harpender stadiometer and body weight with a standard electric scale. BMI was calculated as weight divided by height squared (kg/m^2^). The participants were further imaged with a Lunar Prodigy or iDXA scanner (GE Medical Systems; Software version 9.20.122-9.30.044) to collect hip DXA images. The short-term precision was determined from duplicate scans of 14 adult subjects and was 1.6% for FN aBMD, 1.6% for BMC, and 1.7% for bone area. The grip strength of the dominant hand was tested with a Jamar hydraulic hand dynamometer (5030J). Maximum knee extension torque from isokinetic knee flexion and extension was tested for the right leg with a Biodex System III Pro (Biodex Medical Systems Inc.) dynamometer at speeds of 60° and 180° per second. The maximum jump height was calculated based on the time of flight^39^ of each participant using the time-it basic set (Monark AB). Three attempts were recorded for all these tests, from which the best performance was selected for this study. There were seven men with insufficient/missing DXA scans, resulting in 1094 individuals included in this study. The hours of physical activity, excluding walking, were available from questionnaire data but were not used in this study since the answers provided included several unlikely estimates (eg, one-third of the subjects reported over 20 h/wk of intense physical activity).

**Figure 1:**
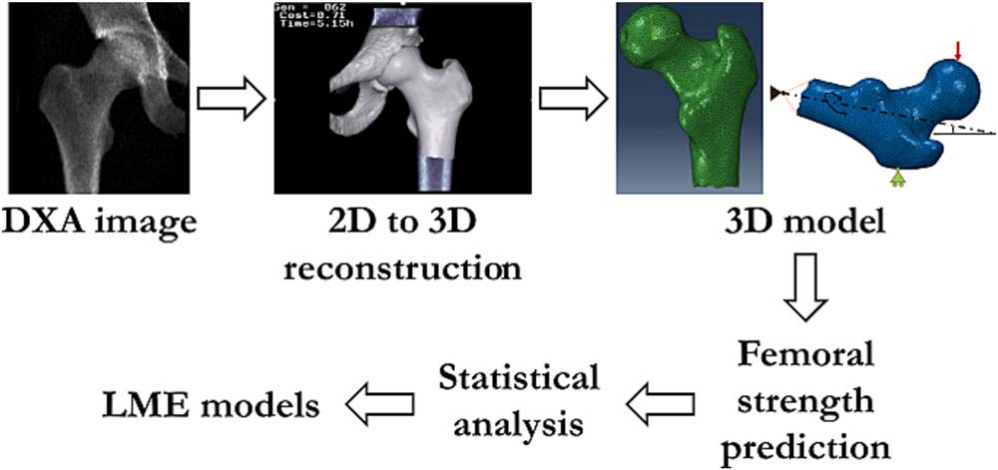
General description of the approach. The 2D-to-3D reconstruction method starts with the identification of landmarks on the 2D DXA image using an automated tool. These landmarks are then used in the merit function to reduce the cost using a genetic algorithm. The genetic algorithm also reduces the sum of the absolute difference between the densities of the omega region between the SSAM and the 2D image, and mesh quality.^37^ The obtained 3D meshed model is then simulated in 10 different sideways fall boundary conditions by varying the internal rotation and adduction angle between 0 and 30°.^22^^,^^40^^,^^50^ Femoral strength was predicted using a previously validated principal strain criterion using Abaqus CAE and an in-house MATLAB script.^22^^,^^51^

Subject-specific FE models were created to analyze proximal femoral strength in the MrPeak cohort and linear mixed-effect models (LMEs) were used to identify parameters that could explain the most variation in FE-predicted proximal femoral strength (Figure 1). Three-dimensional FE models of the proximal femur were obtained for each 2D-DXA scan using a previously developed pipeline based on statistical shape and appearance models (SSAMs).^37^^,^^38^ The SSAMs of a proximal femur and a hemipelvis were fitted to the 2D DXA image of the right hip for each subject by varying their principal components scores through a genetic optimization algorithm that minimized the sum of the absolute differences between the target DXA image and the simulated radiograph obtained by projecting the SSAM instances. This procedure is described in detail in Väänänen et al.^37^ and Grassi et al.,^29^^,^^34^ and it results in a subject-specific 3D FE model of the proximal femur. These FE models were then used to estimate femoral strength using linear elastic simulations and a validated asymmetric maximum principal strain criterion.^40^ Femoral strength was predicted for 10 different configurations resembling a fall to the side by varying the adduction and internal rotation angles between 0 and 30° at regular intervals of 10° in a full factorial scheme. The maximum femoral strength predicted across the 10 loading configurations was selected as the subject’s femoral strength. The underlying methods have been previously presented,^29^^,^^37^ and validated in terms of femoral strength against ex vivo mechanical tests,^34^^,^^38^ as well as against prospective clinical cohorts of elderly men in terms of the prediction of incident hip fractures.^22^ Femoral neck volume was calculated for each of the reconstructed 3D FE models of the proximal femur by summing the volume of each tetrahedral element in the FN of the meshed femur (MATLAB version: 24.1.0, the MathWorks, Inc.).

The minimum redundancy and maximum relevance algorithm and F-test algorithm were used to pre-screen the parameters, which were later tested individually in linear mixed effect (LME) models to identify the influence of each parameter.^41^ Out of all the parameters obtained from the MrPeak cohort, FN aBMD, BMC, BMI (kg/m^2^), age, height and weight, FN volume and area, scanner ID, month of scan, jump height, maximum knee extension torque from the isokinetic muscle test, and grip strength were evaluated for their effect on FE-predicted proximal femoral strength. Since the subjects in the MrPeak cohort were imaged with either a Lunar Prodigy or Lunar iDXA scanner, the scanner ID was added as a fixed effect in the LME models to account for the effects of possible systematic differences between scanners (aBMD measurements were cross-calibrated, but subject-specific FE models may be affected by changes in the image spatial resolution and signal-to-noise ratio^37^). Linear mixed effect models^42^ were created to identify the combinations of parameters that could explain most of the variations in FE-predicted proximal femoral strength (MATLAB version: 24.1.0, the MathWorks, Inc.). Subjects that did not participate in any muscle function tests (*n* = 50) were excluded from LME analysis, resulting in a cohort with 1044 subjects.

Age and month of scan were considered as categorical effects to investigate the influence of the subject’s age and seasonal variation on aBMD and the FE-predicted proximal femoral strength.^43^ An optimal set of parameters that explained most of the variation in FE-predicted proximal femoral strength was selected based on the coefficient of determination (*R*^2^) and the statistical significance. Jump height, grip strength, and maximum knee extension torque from the isokinetic leg muscle strength test were added as fixed effects in each of these models.

Mean and 95% CI plots were created to analyze the influence of age on FE-predicted proximal femoral strength and FN aBMD. Linear mixed-effect models were used to study the effect of various parameters on the FE-predicted proximal femoral strength. The relationship between FE-predicted proximal femoral strength and FN area with the subject’s weight, and height was analyzed for the total cohort and for normoweight subjects only. For the purpose of this study, we defined normoweight as a BMI range that included normal weight, moderate thinness and overweight according to WHO definitions (BMI range 17 ≤ BMI < 30). Pearson’s correlation was used to determine the correlation between various parameters using a linear regression analysis.

The measured performance in terms of jump height, grip strength, and maximum knee extension torque tests were discretized into bins of uniform width (10 cm for jump height, 12 kg for grip strength, and 70 Nm for torque). Finite element-predicted proximal femoral strength was compared with jump height, maximum knee extension torque data from the isokinetic muscle test, and grip strength test to analyze the relationship between various strength tests and FE-predicted proximal femoral strength.

## Results

**Figure 2:**
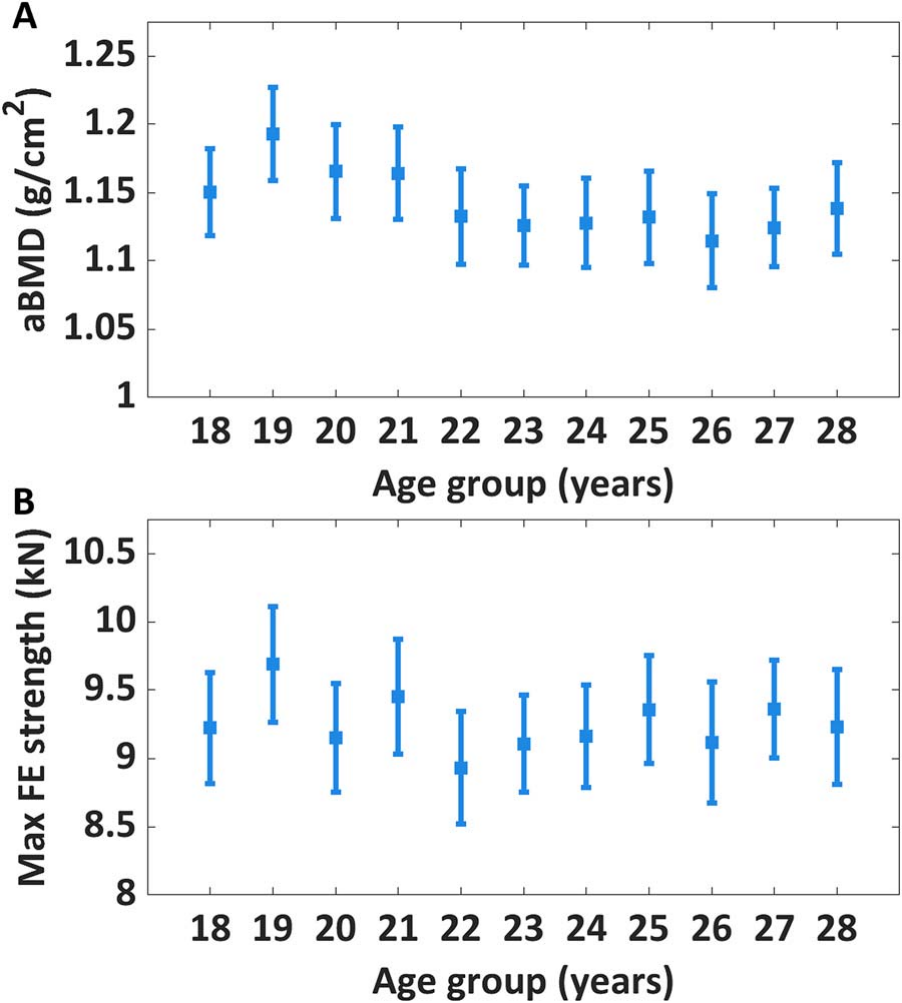
Mean FN aBMD (A) and FE-predicted proximal femoral strength (B). Data is presented as means with 95% CIs. There was a statistically significant inverse relation between the age groups from age 18-28 in FN aBMD (*r* = −0.10; *p* = .001) but not FE-predicted proximal femoral strength (*r* = −0.02; *p* = .55).

There was a statistically significant negative correlation between age and FN aBMD among the 1-yr age groups in men between the ages of 18 and 28 yr (*r* = −0.10, *p* = .001), which is consistent with previous reports on the same cohort.^5^ In contrast, we did not observe any significant correlation between FE-predicted proximal femoral strength and the different 1-yr age groups (*r* = −0.02, *p* = .55) (Figure 2 and Table 1). No statistically significant correlation was found between the FN area and 1-yr age groups (*r* = 0.05, *p* = .06). Table 2 shows the characterization of MrPeak cohort according to the 1-yr age groups.

**Table 1:** A univariate comparison of correlation coefficient and *p*-value for all measurements with respect to FE-predicted proximal femoral strength of all subjects and subjects in the BMI range of 17 ≤ BMI < 30 kg/m^2^ referred to as *r*_all_, *p*_all_, *r*_normo,_ and *p*_normo_, respectively. The statistical significance (alpha) after applying the Bonferroni correction was 0.0042 (0.05/12 = 0.0042).

**Factors**	**FN aBMD**	**FN BMC**	**FN area**	**FN volume**	**height**	**weight**	**age**	**Jump** **height**	**Grip strength**	**MKE torque**	**Acquisition month**	**Scanner ID**
***r***_**all**_	0.85	0.86	0.29	0.26	0.23	0.37	−0.01	0.12	0.24	0.35	0.04	−0.13
***P***_**all**_	<0.001	<0.001	<0.001	<0.001	<0.001	<0.001	0.55	<0.001	<0.001	<0.001	0.20	<0.001
***r***_**normo**_	0.84	0.85	0.30	0.26	0.22	0.37	−0.01	0.15	0.24	0.34	0.04	−0.13
***P***_**normo**_	<0.001	<0.001	<0.001	<0.001	<0.001	<0.001	0.65	<0.001	<0.001	<0.001	0.24	<0.001

**Table 2:** The MrPeak cohort characterized according to age, subject’s height, body weight, BMI, FN BMC, FN aBMD, FN area, and FE-predicted proximal femoral strength. Data is presented as mean (SD).

**Age (years)**	**18**	**19**	**20**	**21**	**22**	**23**	**24**	**25**	**26**	**27**	**28**
**Number of subjects**	98	99	94	103	101	107	87	98	98	118	91
**Height** **(cm)**	181.7 (7.2)	181.3 (7.6)	181.1 (7.3)	181.9 (6.7)	181.9 (6.7)	180 (6.4)	181.2 (6.9)	180.7 (6.1)	181.9 (6.8)	180.9 (7.3)	182.1 (6.7)
**Weight (Kg)**	74.8 (12.9)	76.5 (12.1)	78.3 (13.5)	80.9 (15.6)	80 (13.7)	75.4 (10.5)	78.4 (12.9)	79.8 (9.9)	82.4 (15.1)	80.3 (13.3)	82.8 (12.8)
**BMI** **(kg/m**^**2**^**)**	22.6 (3.5)	23.2 (3.1)	23.8 (3.4)	24.3 (4.2)	24.2 (4.2)	23.2 (3.1)	23.8 (3.6)	24.4 (2.9)	24.9 (4.6)	24.5 (3.6)	24.8 (3.4)
**FN BMC (g)**	6.39 (1.17)	6.62 (1.11)	6.43 (1.05)	6.5 (1.13)	6.28 (1.11)	6.17 (0.97)	6.27 (0.95)	6.37 (1.13)	6.24 (1.17)	6.25 (1.05)	6.43 (1.04)
**FN aBMD** **(g/cm** ^**2**^ **)**	1.15 (0.16)	1.19 (0.17)	1.17 (0.17)	1.17 (0.17)	1.13 (0.18)	1.13 (0.15)	1.13 (0.15)	1.13 (0.17)	1.11 (0.17)	1.12 (0.16)	1.4 (0.16)
**FN area (cm** ^**2**^ **)**	5.54 (0.49)	5.54 (0.41)	5.52 (0.41)	5.57 (0.50)	5.54 (0.42)	5.48 (0.41)	5.57 (0.38)	5.61 (0.39)	5.59 (0.46)	5.56 (0.39)	5.64 (0.35)
**FE strength (kN)**	9.22 (2.03)	9.69 (2.11)	9.15 (1.94)	9.48 (2.15)	8.93 (2.07)	9.10 (1.85)	9.16 (1.76)	9.36 (1.98)	9.11 (2.21)	9.36 (1.97)	9.24 (2.01)

**Figure 3:**
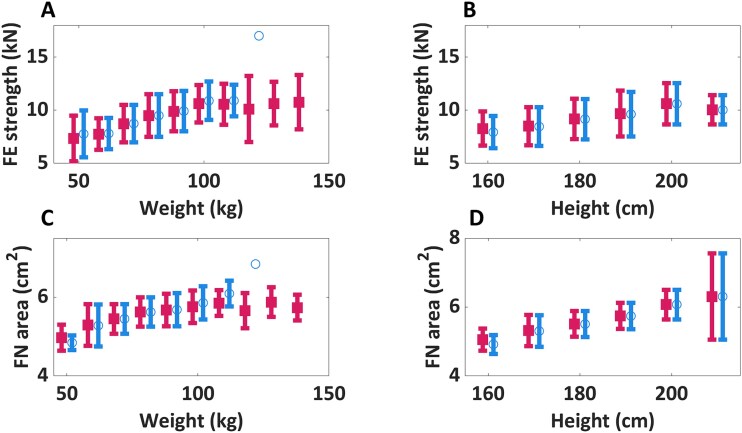
Max FE-predicted proximal femoral strength and FN area as a function of the subject’s height and weight. Data is presented as mean (circles for data in blue, squares for data in magenta) and standard deviation (error bars). Subjects were assigned to binned groups with interval size of 10 kg for weight and 10 cm for height (example: 45-55 kg for weight and 155-165 cm for height). Squares in magenta include all subjects, and circles in blue include subjects in the BMI range of 17 ≤ BMI < 30 kg/m^2^. Statistically significant higher FE-predicted proximal femoral strength and FN area were observed with higher height (*p* < .001) and weight (*p* < .001) for both groups of subjects.

In a linear regression analysis, we found that the FE-predicted proximal femoral strength positively correlated with body weight (*r* = 0.35, *p* < .001) and body height (*r* = 0.22, *p* < .001) for all subjects (Figure 3 and Table 1). Moreover, the FN area showed a positive correlation with body weight (*r* = 0.31, *p* < .001) and body height (*r* = 0.41, *p* < .001) for all subjects (Figure 3, Figure S1). When conducting the same analysis only with the men in the BMI range of 17 ≤ BMI < 30 kg/m^2^, the correlation coefficients increased (Δ*r* = 0.01-0.05) (Figure 3, Figure S2).

**Figure 4:**
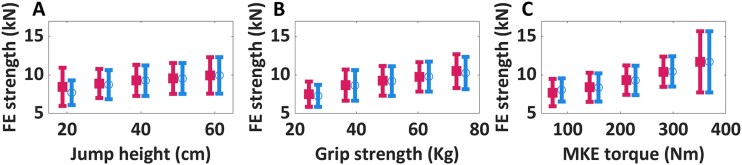
Higher jump height (A), grip strength (B), and maximum knee extension torque (C) may be related to higher max FE-predicted femoral strength. Data is presented as mean and standard deviation. Squares in magenta include all subjects, and circles in blue include subjects in the BMI range of 17 ≤ BMI < 30 kg/m^2^. The bin centers in the jump height, grip strength, and MKE torque start at 20 cm, 26 kg, and 80 nm, respectively, with a bin width of 10 cm,12 kg, and 70 nm. Statistically significant higher FE-predicted proximal femoral strength was observed with higher jump height (*p* < .001), grip strength (*p* < .001), and maximum knee extension torque (*p* < .001) for both groups of subjects. A relative percentage difference of 12%, 20%, and 23% in proximal femoral strength between the group with the worst performance (inferior values) and the group with the best performance (superior values) was observed for jump height, grip strength, and maximum knee extension torque, respectively. A relative percentage difference of 23%, 29%, and 31% in proximal femoral strength between the group with the worst performance (inferior values) and the group with the best performance (superior values) was observed for jump height, grip strength, and maximum knee extension torque, respectively, for subjects in the BMI range of 17 ≤ BMI < 30 kg/m^2^.

There was a positive correlation between FE-predicted proximal femoral strength and groups divided according to jump height (*r* = 0.13, *p* < .001), grip strength (*r* = 0.23, *p* < .001), and maximum knee extension torque (*r* = 0.31, *p* < .001), respectively (Figure 4 and Table 1). When conducting the same analysis by only including men in the BMI range of 17 ≤ BMI < 30 kg/m^2^, the correlation coefficients increased for jump height (Δ*r* = 0.02, Figure 4).

**Figure 5:**
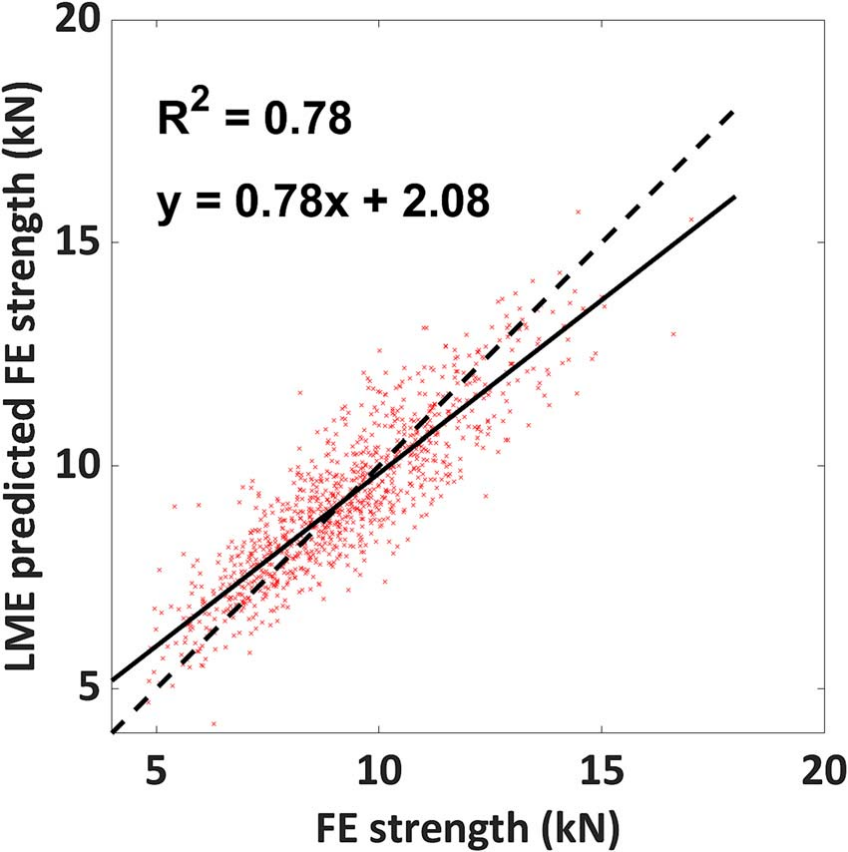
The LME prediction of FE-predicted proximal femoral strength versus FE-predicted proximal femoral strength plot. The *R*^2^ value shows the goodness of fit for the linear regression analysis performed on the two variables. The y-axis of the plot is based on LME eqn (1), which consists of FN aBMD, FN area, height, and scanner ID.

Based on the univariate comparison of the correlation coefficient and *p*-value of various parameters with FE-predicted proximal femoral strength, parameters were added to the LME model with FN aBMD/BMC to identify the best coefficient of determination. Equations 1 and 2 represent the LME models that had the highest coefficient of determination:


(1) \begin{eqnarray*}&& \text{FE-predicted proximal femoral strength} \sim \textrm{FN aBMD}\nonumber\\ && \quad + \text{FN area + subject's height + scanner} + \mathrm{ID}\end{eqnarray*}

(2) \begin{eqnarray*}&& \text{FE-predicted proximal femoral strength} \sim \textrm{FN BMC}\nonumber\\&& + \text{FN area + subject's height + scanner} \mathrm{ID}\end{eqnarray*}

where the left-hand side (ie, before the ~) specifies the response variable, and the right-hand side the predictor variables. The LMEs model including FN aBMD, FN area, height, and scanner ID (eqn 1, Table S3) could explain 78% of the variation in FE-predicted proximal femoral strength (Figure 5). An identical result (*R*^2^ = 0.78) was obtained when FN BMC replaced FN aBMD (eqn 2, Table S4) in the model. FN aBMD, FN BMC, FN area, and height could separately explain ~71%, ~72%, 8.8%, and 5.5% of the variation in FE-predicted proximal femoral strength, respectively.

In a univariate LME model, the subject’s weight explained 13.7% (*r* = 0.37, *p* < .001) of the variance in FE-predicted proximal femoral strength, jump height 1.6% (*r* = 0.12, *p* < .001), grip strength 5.7% (*r* = 0.24, *p* < .001), and maximum knee extension torque 11.9% (*r* = 0.35, *p* < .001). However, none of these traits improved the *R*^2^ value in any of the two models. Finite element-predicted proximal femoral strength was positively correlated with FN BMC, FN aBMD, subject’s weight, height, FN volume, FN area, jump height, BMI, grip strength, and maximum knee extension torque. Linear mixed effect models did not show any dependence of FE-predicted proximal femoral strength on age (*R*^2^ = 0).

## Discussion

This study aimed to determine whether proximal femoral strength and aBMD decreased with age in a population-based cohort of young adult men and which parameters could best explain the inter-individual variation in proximal femoral strength. We found FN aBMD peaked in the 19-yr age group and be ~5% lower for the 28-yr age group (*p* = .001, Figure 2), consistent with previous reports on the same cohort.^5^ We hypothesized that proximal femoral strength would instead not change significantly across the one-year age groups, and that variations in FN area, patients’ anthropometrics and indicators of physical performance would compensate for the variations in FN aBMD.

We indeed found that proximal femoral strength did not change significantly for the examined cohort of men in the age range of 18-28 yr. However, FN area also did not increase significantly with age in the examined age span. This suggests that variations in FN area may not be the sole factor that explains why femoral strength does not decline alongside FN aBMD at age 19. When we looked at the relationship between both FE-predicted proximal femoral strength and FN area as a function of the subjects’ anthropometrics, we observed a positive but moderate correlation with weight (*r* = 0.35 and 0.31, respectively) and height (*r* = 0.22 and 0.41, respectively). This correlation became stronger (Δ*r* = 0.01-0.05, Figure 3A-C) when we excluded underweight and obese subjects. Such results make sense from a biomechanical perspective and are consistent with previous findings.^44^^,^^45^ Still, the weak to moderate correlations suggested that other factors may play a role in determining variations in proximal femoral strength.

When we looked at the association between proximal femoral strength and indicator of physical performance, we observed that higher values in jump height, grip strength, and maximum knee extension torque were associated with higher FE-predicted proximal femoral strength (Figure 4). This was also consistent with previous findings,^46^^,^^47^ and generally corroborates the established notion of bone adaptation in response to loading and mechanical stimuli. Notable from our measurements was that simple measurements of grip strength were equally well associated (*r* = 0.23) with proximal femoral strength as more complex and expensive assessments such as jump height and knee extension torque (*r* = 0.13 and 0.31, respectively).

The LME analysis did not show any difference in FE-predicted proximal femoral strength between the different 1-yr age groups, which again supported our initial hypothesis. Simple LME models accounting for aBMD (or BMC) together with FN area and the subject’s height (and scanner ID as a categorical variable) could explain 78% of the variation in proximal femoral strength. Linear mixed effect models with FN aBMD and BMC produced similar *R*^2^ values, pointing toward the similarity of their contributions when paired with FN area, subject’s height, and scanner ID. The LME model showed a small systematic bias, as slope and intercept were different from unity (0.78) and zero (2.08 kN), respectively This does not, however, invalidate the main result that the LME model can explain 78% of the variability in FE-predicted proximal femoral strength, which is a substantial improvement compared to what could be explained by any of the individual predictors alone (Figures S1 and S2). Even though jump height, grip strength, and maximum knee extension torque showed positive correlation coefficients with FE-predicted proximal femoral strength, their interactions with FN aBMD, FN BMC, FN area, subject’s height, and scanner ID could be the reason for the unchanged *R*^2^ value of the LME models. Adding jump height, grip strength, and maximum knee extension torque to the LME models could have introduced multicollinearity, which might explain the unchanged coefficient of determination. The coefficient of determination of individual variables is not additive and the LME models helped us to identify the optimum set of parameters that could explain the variations in each model.

Sideways fall FE simulations were conducted to evaluate the FE-predicted proximal femoral strength of each femur to counter the possible inaccuracies arising by using aBMD as a surrogate for femoral strength in growing adults. We simulated 10 different loading conditions and selected the maximum FE-predicted proximal femoral strength for the statistical analysis, since we aimed at investigating peaks in bone mass and bone strength. The aBMD results showed a relative difference of approximately −6% between the 19 and 26-yr age groups (Figure 2). This was in line with what was reported by Høiberg et al. in a cohort of 783 young men where aBMD was seen to undergo a decline of about 5% from the age of 22 until the age of 30.^10^ Similarly, Sagelv et al., Berger et al., and Lindgren et al. also reported a decline in FN aBMD starting from the age of 19 yr.^5^^,^^9^^,^^11^ The peak of FN aBMD in the MrPeak cohort matched with other studies on similarly aged cohorts.^9^^,^^11^ Despite these changes in aBMD, we observed that overall proximal femoral strength did not change significantly between the ages of 18 and 28 yr. These results support the findings of Keaveny et al., who found that the proximal femoral strength predicted by FE analysis does not start to decline considerably until the age of 40 in women and 50 in men.^35^ Compared to that study, the present one was carried out specifically in the age range where FN aBMD has been reported to start declining. Higher grip strength of the dominant hand and higher maximum knee extension torque from the isokinetic muscle test of the lower leg in our study showed a positive correlation with FE-predicted proximal femoral strength, which was also observed in previous studies where aBMD was used as a predictor of bone strength.^12^^-^^14^ However, the correlation between grip strength, jump height, and maximum knee extension torque and FE-predicted proximal femoral strength showed weak associations (*r* = 0.12-0.35) between various strength tests and their ability to predict strength in distal weight-bearing anatomical sites.

We used a previously proposed method developed by our research group to obtain 3D subject-specific FE models from 2D DXA scans of the hip and predict proximal femoral strength.^37^ Our method has a similar accuracy in reconstructing 3D anatomies from DXA scans (mean shape accuracy ~1 mm, errors in vBMD ~10%) to the commercially available 3D-Shaper.^48^ Additionally, our method has been validated in terms of accuracy in predicting proximal femoral strength against both ex vivo mechanical tests and CT-based FE models.^34^^,^^38^ Recently, it was also found to predict the risk of incident hip fracture better than aBMD in prospective cohorts of elderly men and women.^22^^,^^29^ The method is fully automated, which allowed us to reconstruct all the femurs in this study within ~5 wk. The subject-specific 2D-to-3D reconstructions took ~6.6 h per reconstruction on a 2.67 GHz 24-core supercomputing facility.

One limitation of this study is that it was based on a cross-sectional cohort, which makes it impossible to draw conclusions about longitudinal changes. Still, the cross-sectional cohort used in this study was designed specifically to investigate the occurrence of PBM in young adult men and included more than 87 subjects within each one-year age group for which extensive information, including densitometric information, questionnaire data, and strength tests, were available. Another limitation is that the SSAM used in this study was not specifically trained on subjects within this age range (40 men, 19 women, median age 58 yr and range 18-88 yr,^37^). However, 3D FE models obtained from the same SSAM have been validated, among others,^34^^,^^38^ against ex vivo mechanical tests that included femurs of younger male donors (three samples, including one femur from a 22-yr old donor, FN volumetric BMD 0.6-1.16 g/cm^3^)^38^ as well as simulated biofidelic sideways falls (11 samples with a mean age of 81 yr from donors).^34^ More recently, the method showed its clinical applicability by predicting incident fractures for hundreds of cases in prospective cohorts of men (aBMD >1.0 g/cm^2^, average predicted femoral strength >7 times the subjects’ body weight for the healthy controls)^22^ and women.^29^ The criterion for estimating proximal femoral strength is derived from Schileo et al.^40^ and has been validated on men and women with single-leg stance and sideways fall loading conditions. This method is based on yield strain values for cortical bone, which have been found to remain constant over the years while being insensitive to variations in bone stiffness and strength.^49^ Last, the hours of physical activity obtained from the questionnaires available for this cohort were not completely reliable. This imposed difficulties in establishing associations between proximal femoral strength and amount of physical exercise. We, however, were able to use quantitative measurements to present associations between proximal femoral strength and physical performance (Figure 4).

In conclusion, we found no statistically significant differences in proximal femoral strength between the 1-yr age groups in men aged 18-28 yr. Inter-subject variation in proximal femoral strength were predominantly associated with FN aBMD, body height, FN area, and better outcomes in jump height, grip strength, and maximum knee extension torque tests.

## Supplementary Material

supplementary_material_ziaf136

## Data Availability

Data is available from the corresponding author upon reasonable request.
