# Supplementary material for: Variations over time in proximal femoral strength in young adult men are not explained by areal bone mineral density alone
Source: JBMR Plus. 2025 Aug 16;9(10):ziaf136. doi: 10.1093/jbmrpl/ziaf136 (PMC12445841; doi:10.1093/jbmrpl/ziaf136)
Supplement: supplementary_material_ziaf136 [file supplementary_material_ziaf136.docx]

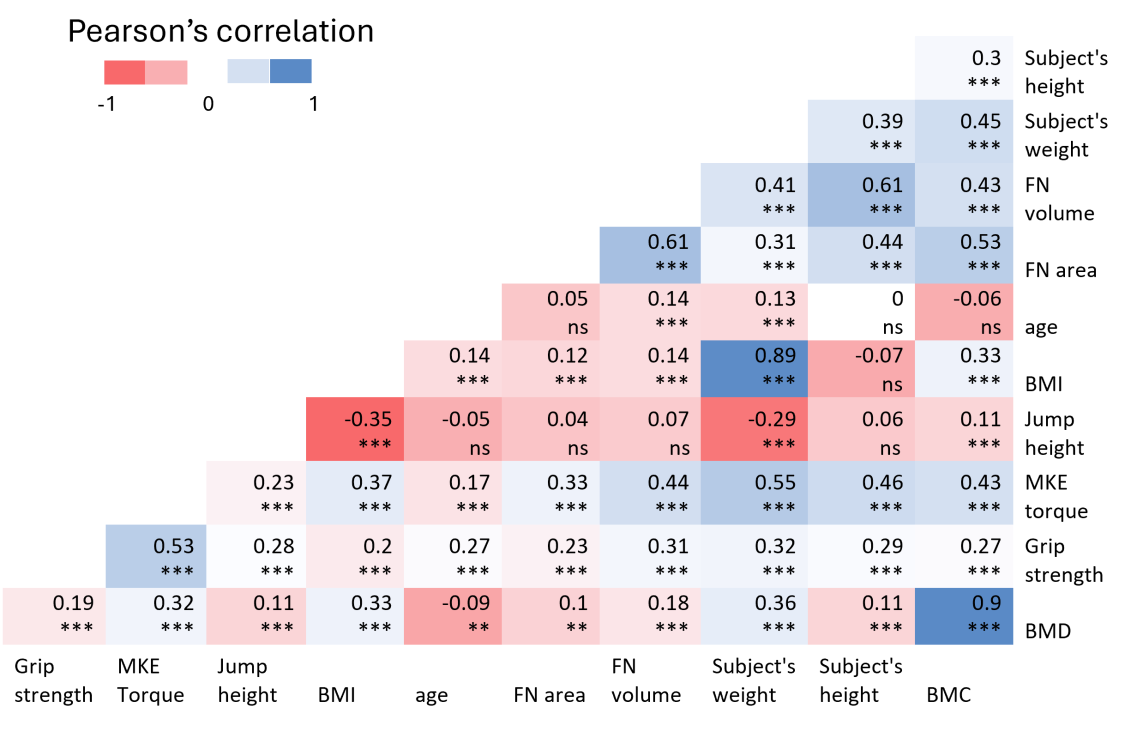
 Figure S1: Comparison of the coefficient of correlation and significance level between parameters of LME models shown as a heatmap for all subjects. The statistical significance (alpha) after applying the Bonferroni correction was 0.0055 (0.05/9=0.0055). In the above figure, ‘ns’ means non-significant, ‘*’ means p <0.0055, '**’ means p <0.001, and ‘***’ means p<0.0001.


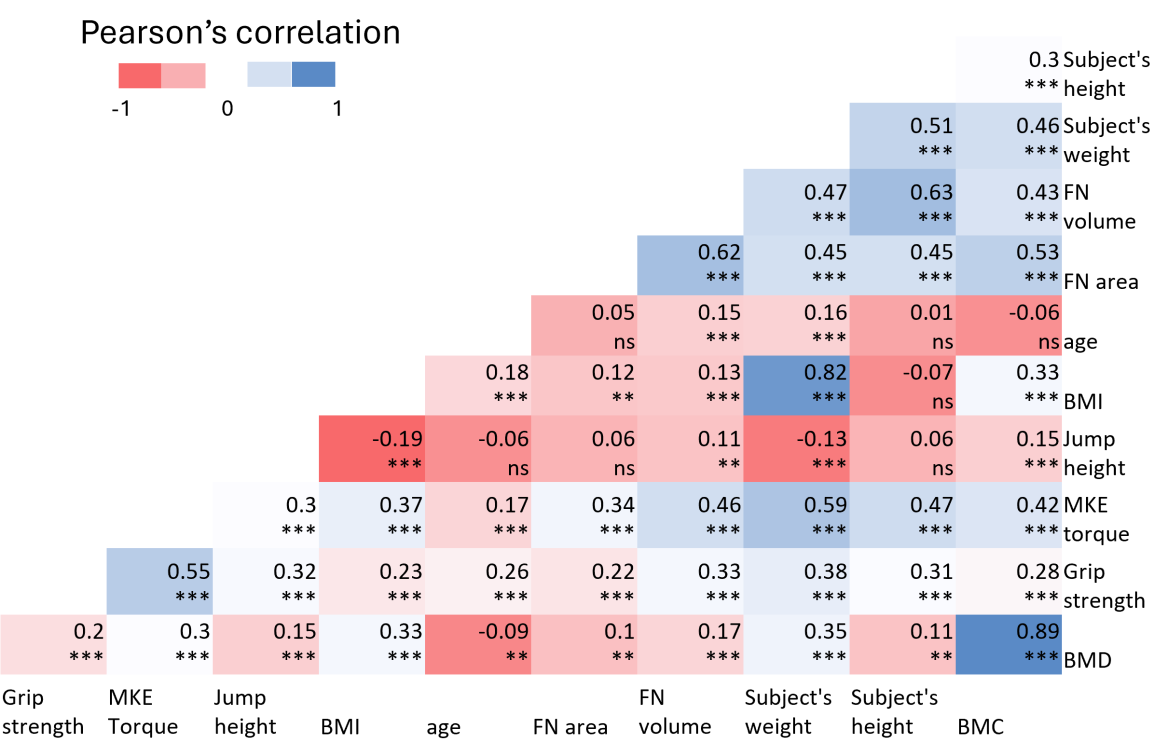


Figure S2: Comparison of the coefficient of correlation and significance level between parameters shown as a heatmap for subjects in the BMI range of 17≤BMI<30 kg/m^2^. The statistical significance (alpha) after applying the Bonferroni correction was 0.0055 (0.05/9=0.0055). In the above figure, ‘ns’ means non-significant, ‘*’ means p <0.0055, '**’ means p <0.001, and ‘***’ means p<0.0001.

**
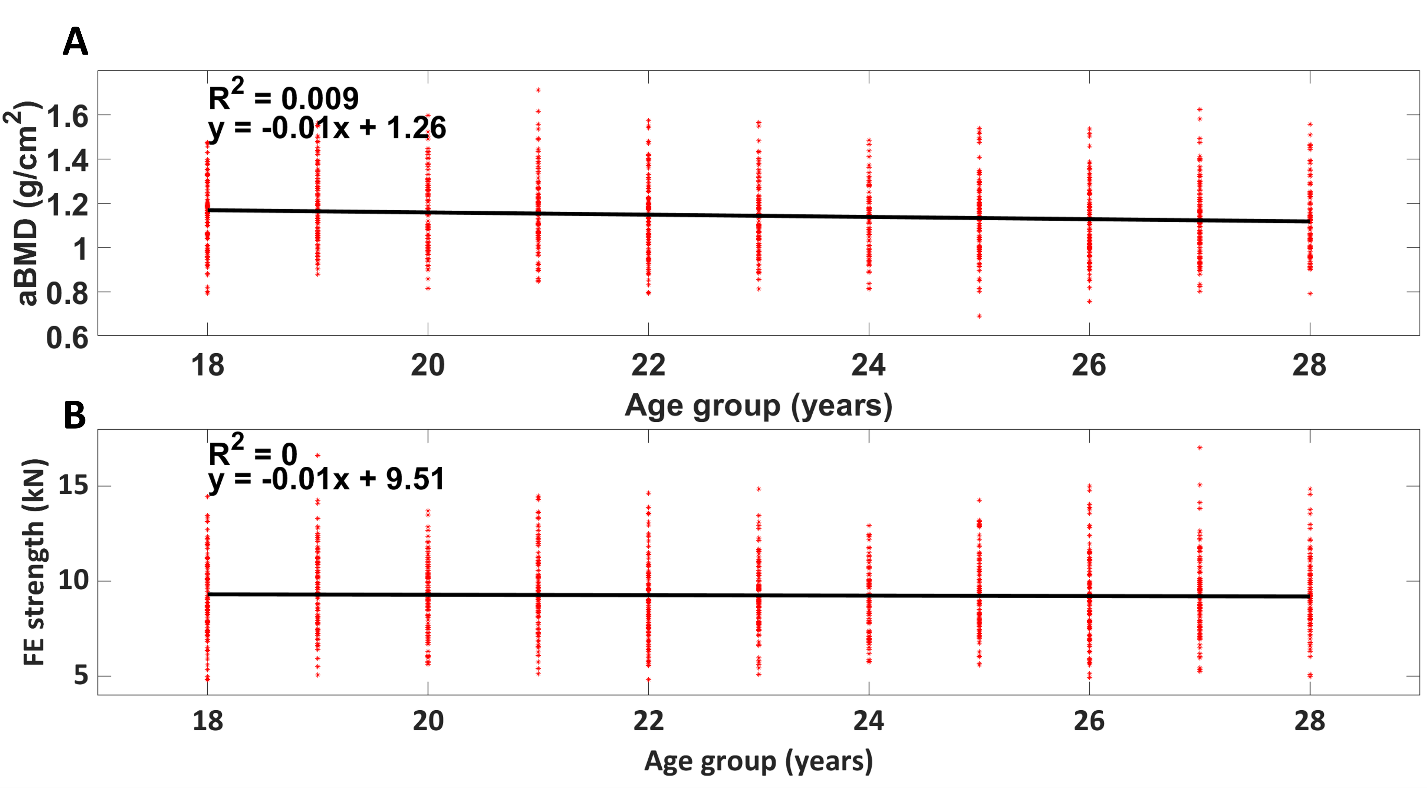
**

Figure S3: A scatter plot with coefficient of determination, slope, and intercept for Figure 2 (age groups vs aBMD (A) and age groups vs FE strength (B).

**
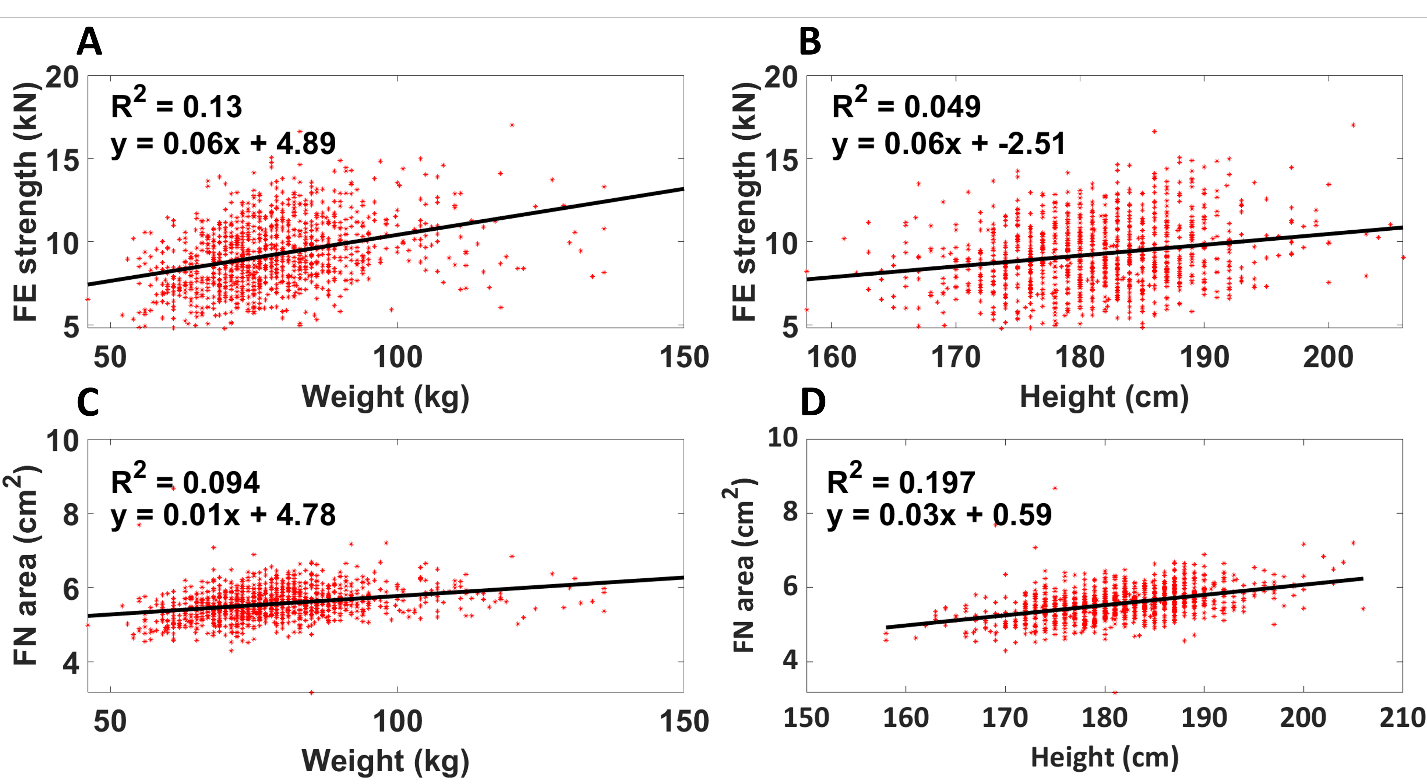
**

Figure S4: A scatter plot with coefficient of determination, slope, and intercept for Figure 3 for all subjects.

**
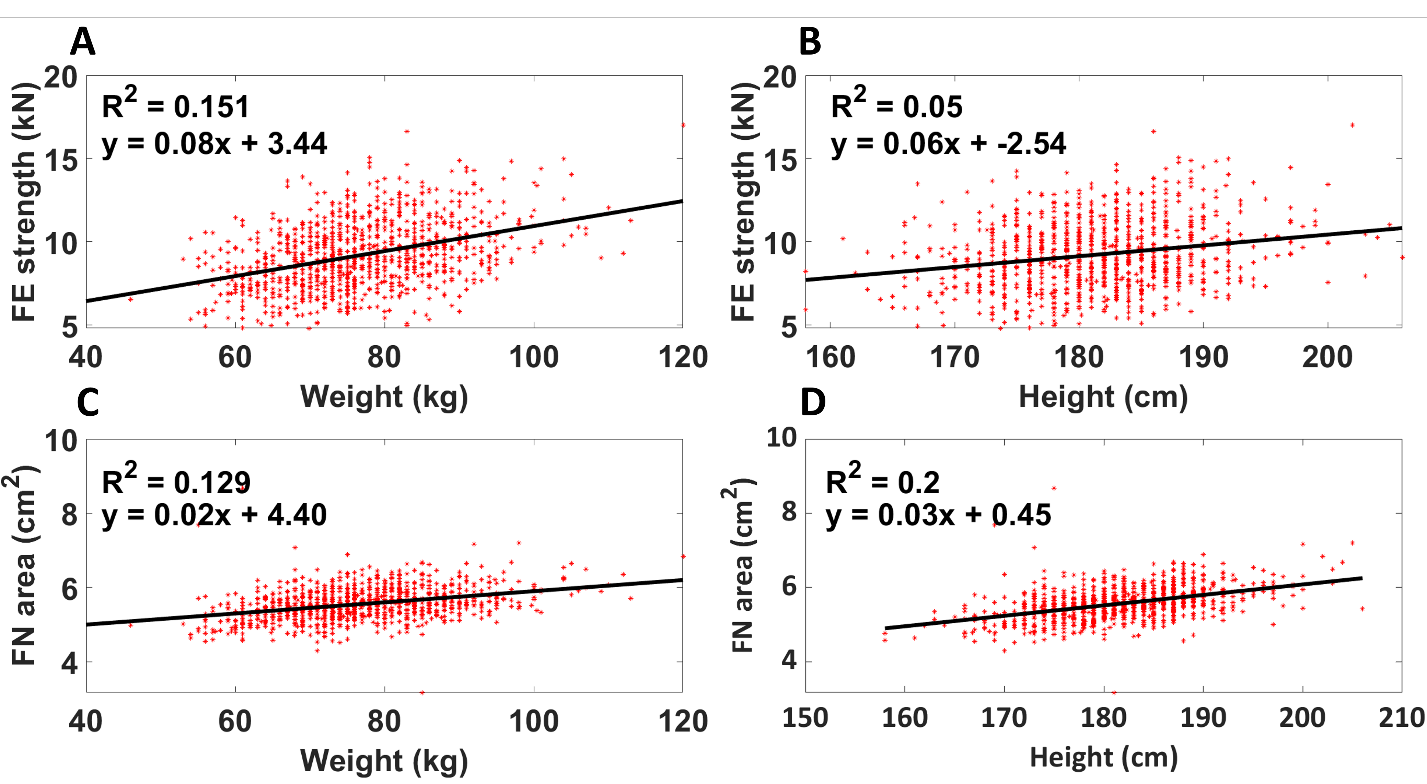
**

Figure S5: A scatter plot with coefficient of determination, slope, and intercept for Figure 3 for subjects in the BMI range of 17≤BMI<30 kg/m^2^.

**
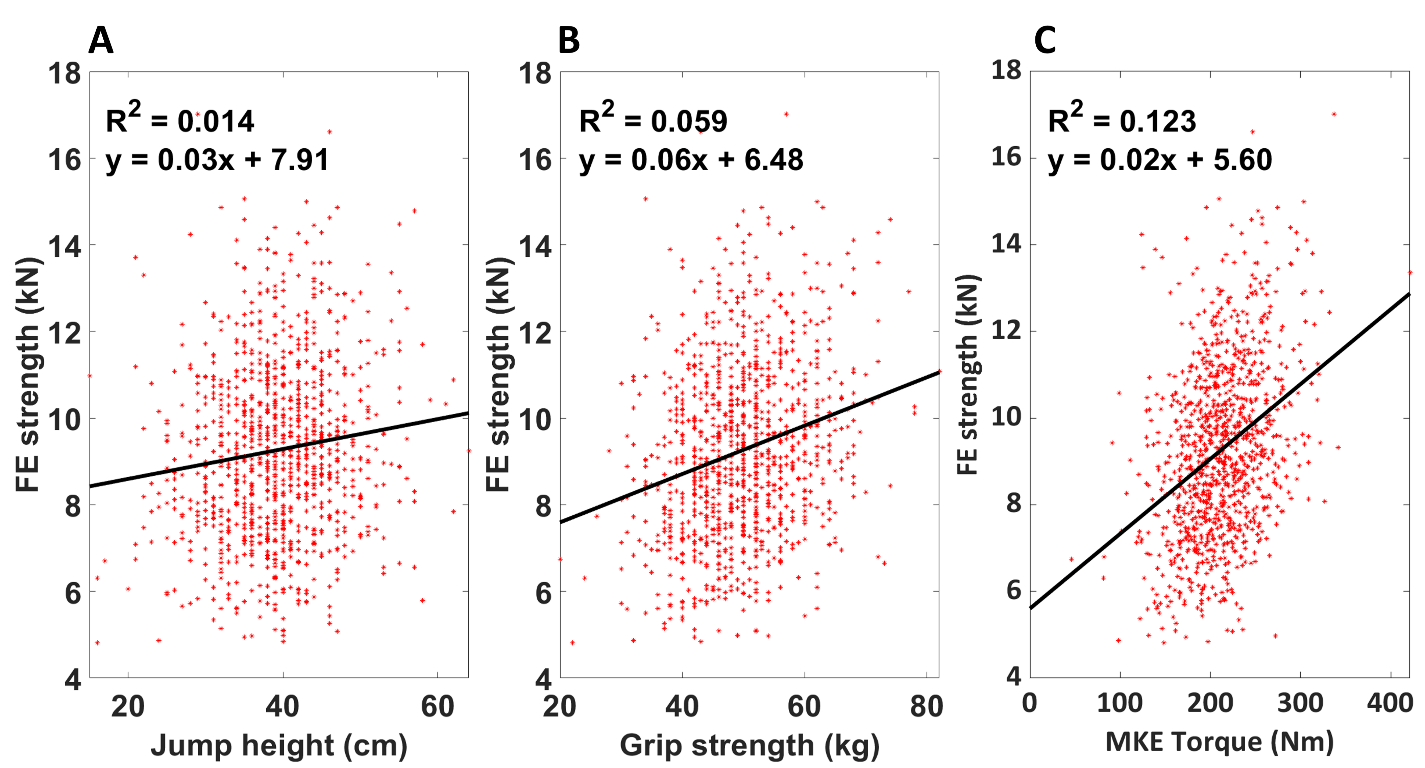
**

Figure S6: A scatter plot with coefficient of determination, slope, and intercept for Figure 4 for all subjects.

**
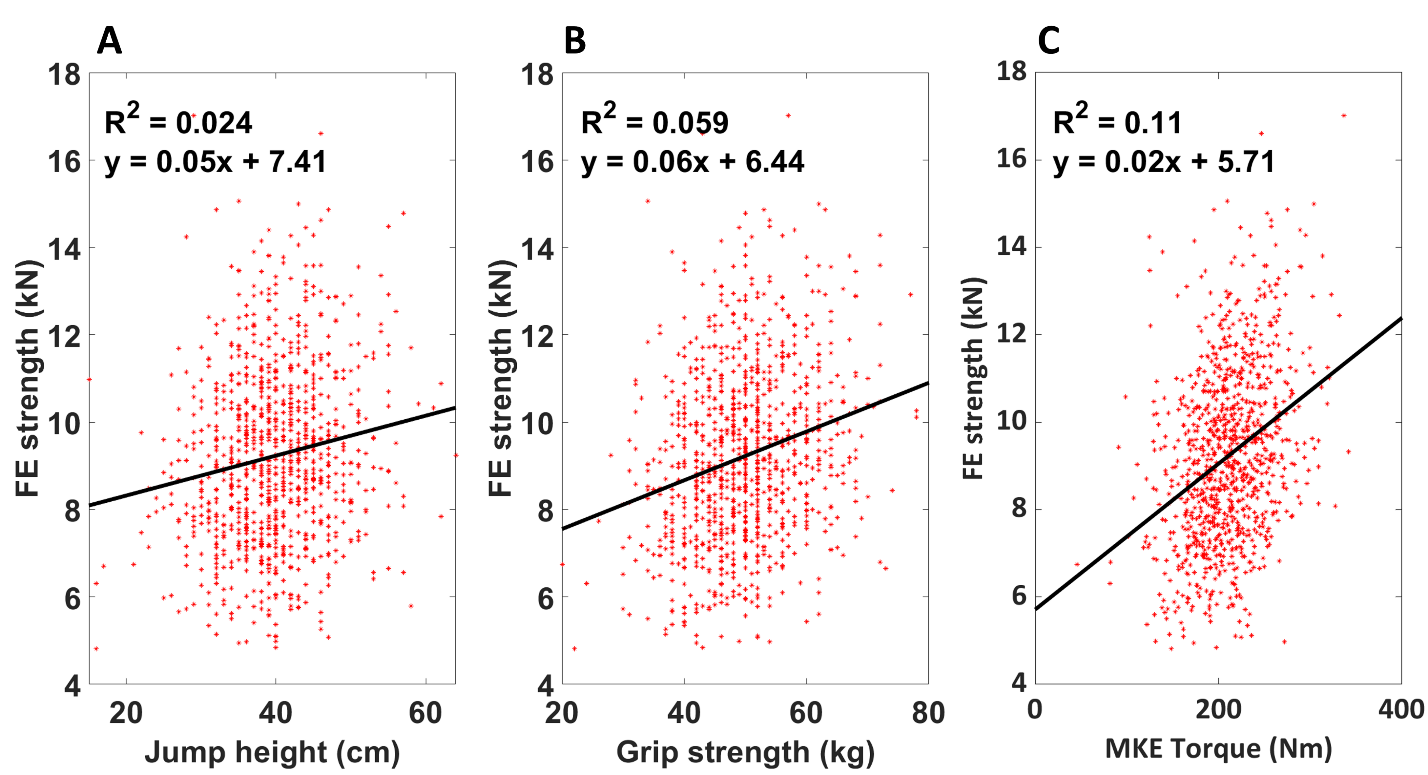
**

Figure S7: A scatter plot with coefficient of determination, slope, and intercept for Figure 4 for subjects in the BMI range of 17≤BMI<30 kg/m^2^.

|  |  | **Estimate** | **Standard error (SE)** | **p-value** |
| --- | --- | --- | --- | --- |
| **FE max vs age** | **Intercept** | 9.51 | 0.45 | <0.001 |
|  | **predictor** | -0.011 | 0.019 | 0.56 |
| **FE max vs body weight** | **Intercept** | 7.17 | 0.18 | <0.001 |
|  | **predictor** | 0.53 | 0.042 | <0.001 |
| **FE max vs body height** | **Intercept** | 7.4 | 0.25 | <0.001 |
|  | **predictor** | 0.58 | 0.08 | <0.001 |
| **FE max vs jump height** | **Intercept** | 8.24 | 0.249 | <0.001 |
|  | **predictor** | 0.342 | 0.081 | <0.001 |
| **FE max vs grip strength** | **Intercept** | 7.46 | 0.24 | <0.001 |
|  | **predictor** | 0.588 | 0.077 | <0.001 |
| **FE max vs MKE torque** | **Intercept** | 6.4 | 0.27 | <0.001 |
|  | **predictor** | 0.986 | 0.092 | <0.001 |
| **FN area vs body weight** | **Intercept** | 5.17 | 0.038 | 0 |
|  | **predictor** | 0.098 | 0.009 | <0.001 |
| **FN area vs body height** | **Intercept** | 4.82 | 0.051 | 0 |
|  | **predictor** | 0.23 | 0.016 | <0.001 |
| **FN aBMD vs age** | **Intercept** | 1.26 | 0.036 | <0.001 |
|  | **predictor** | -0.005 | 0.001 | 0.001 |

Table S1: Linear regression estimate, standard error, and p-value for all subjects.

|  |  | **Estimate** | **Standard error (SE)** | **p-value** |
| --- | --- | --- | --- | --- |
| **FE max vs age** | **Intercept** | 9.43 | 0.46 | <0.001 |
|  | **predictor** | -0.009 | 0.019 | 0.56 |
| **FE max vs body weight** | **Intercept** | 6.59 | 0.21 | <0.001 |
|  | **predictor** | 0.70 | 0.054 | <0.001 |
| **FE max vs body height** | **Intercept** | 7.34 | 0.27 | <0.001 |
|  | **predictor** | 0.59 | 0.08 | <0.001 |
| **FE max vs jump height** | **Intercept** | 7.93 | 0.27 | <0.001 |
|  | **predictor** | 0.42 | 0.09 | <0.001 |
| **FE max vs grip strength** | **Intercept** | 7.38 | 0.25 | <0.001 |
|  | **predictor** | 0.60 | 0.08 | <0.001 |
| **FE max vs MKE torque** | **Intercept** | 6.4 | 0.28 | <0.001 |
|  | **predictor** | 0.99 | 0.096 | <0.001 |
| **FN area vs body weight** | **Intercept** | 5.01 | 0.046 | 0 |
|  | **predictor** | 0.14 | 0.012 | <0.001 |
| **FN area vs body height** | **Intercept** | 4.80 | 0.054 | 0 |
|  | **predictor** | 0.24 | 0.017 | <0.001 |
| **FN aBMD vs age** | **Intercept** | 1.25 | 0.037 | <0.001 |
|  | **predictor** | -0.005 | 0.002 | 0.001 |

Table S2: Linear regression estimate, standard error, and p-value for subjects in the BMI range of 17≤BMI<30 kg/m^2^.

|  | **Estimate** | **Standard error (SE)** | **p-value** |
| --- | --- | --- | --- |
| **Intercept** | -9.54 | 0.79 | <0.0001 |
| **Body height** | 0.014 | 0.004 | 0.002 |
| **FN aBMD** | 10 | 0.18 | <0.0001 |
| **FN area** | 0.87 | 0.08 | <0.0001 |
| **Scanner ID** | <0.0001 | <0.0001 | <0.0001 |

Table S3: Regression estimates, standard error, and p-value (95% CI) for Figure 5 (Equation 1).

|  | **Estimate** | **Standard error (SE)** | **p-value** |
| --- | --- | --- | --- |
| **Intercept** | 2.08 | 0.79 | 0.008 |
| **Body height** | 0.011 | 0.005 | 0.014 |
| **FN BMC** | 1.77 | 0.032 | <0.0001 |
| **FN area** | -1.11 | 0.087 | <0.0001 |
| **Scanner ID** | <0.0001 | <0.0001 | <0.0001 |

Table S4: Regression estimates, standard error, and p-value (95% CI) for Equation 2.
